# Effect of Processing Parameters on Recrystallization During Hot Isostatic Pressing of Stellite-6 Fabricated Using Laser Powder Bed Fusion Technique

**DOI:** 10.3390/ma17225500

**Published:** 2024-11-11

**Authors:** Soumya Sridar, Xavier Jimenez, Albert C. To, Wei Xiong

**Affiliations:** 1 Physical Metallurgy and Materials Design Laboratory, Department of Mechanical Engineering and Materials Science, University of Pittsburgh, Pittsburgh, PA 15261, USA; 2 Department of Mechanical Engineering and Materials Science, University of Pittsburgh, Pittsburgh, PA 15261, USA

**Keywords:** CALPHAD, Scheil, grain growth, porosity, M_7_C_3_

## Abstract

Crack-free Stellite-6 alloy was fabricated using the laser powder bed fusion technique equipped with a heating module as the first attempt. Single tracks were printed with a build plate heated to 400 °C to identify the processing window. Based on the melt pool dimensions, two combinations (sample A: 300 W/750 mm/s and sample B: 275 W/1000 mm/s) were identified to print the cubes. The as-printed microstructure comprised FCC-Co dendrites with M_7_C_3_ in the interdendritic region. W-rich M_6_C particles were found in the overlapping regions between the melt pools, matching the Scheil simulations. However, gas pores were observed due to the higher nitrogen and oxygen content of the feedstock requiring hot isostatic pressing (HIP) at 1250 °C and 150 MPa for 2 h. Sample A was partially recrystallized with slightly coarsened M_7_C_3_, while sample B underwent complete recrystallization followed by grain growth along with higher coarsening of the M_7_C_3_ after HIP. The varying recrystallization behavior can be attributed to the difference in residual stresses and grain aspect ratio in the as-built condition dictated by laser power and scanning speed. The microhardness after HIP was slightly higher than its wrought counterpart, indicating no severe impact of post-processing on the properties of Stellite-6 alloy.

## 1. Introduction

Stellite is a class of Co-Cr-based hard-facing alloys that are widely used in marine, automotive, aerospace, as well as oil and gas applications to provide wear resistance in high-temperature or corrosive environments [1]. This alloy is also used to protect stainless steel surfaces in the primary cooling circuit of nuclear power plants due to their high hardness [2]. In addition, Co-Cr-based alloys are used in orthopedic implants such as femoral stems due to their excellent wear resistance and biocompatibility [3]. Several works are available in the literature related to modeling the Tresca stress as a failure criterion with the use of CoCrMo implants as hip joint replacements [4,5,6]. The effect of normal walking activity [4] and body mass index [5] was simulated using the 2D axisymmetric finite element model, and it was compared with other common alloys used as bearings in biomedical applications such as Ti-6Al-4V and stainless steel 316L. Stellite-6, with a nominal composition (in wt.%) of Co–28Cr–4.5W–1.2C, was the first alloy to be developed in the Stellite series by Elwood Haynes in the early 1990s [7]. Elements such as chromium and tungsten contribute to solid solution strengthening, and a greater affinity for carbon with chromium to form carbides such as Cr_7_C_3_ and Cr_23_C_6_ imparts good wear resistance even at high temperatures. The conventional manufacturing techniques employed for fabricating Stellite-6 are casting, forging, powder metallurgy, and coating deposition techniques [1,7,8,9,10,11,12,13]. However, the high content of tungsten renders it difficult to machine, and hence, it is not feasible to produce complex shapes using traditional manufacturing for Stellite-6 alloy.

Additive manufacturing (AM) is a fabrication technique capable of producing complex-shaped parts using layer-by-layer deposition [14]. Since Stellite-6 is a weldable alloy, it is a suitable candidate to be manufactured using AM, and the first work to utilize the powder-based directed energy deposition (DED) AM technique for manufacturing this alloy along with its post-heat treatment was reported by Ren et al. [15]. In 2019, three papers were published on fabricating Stellite-6 using different AM techniques. Traxel and Bandyopadhyay [16] used a powder-based DED technique to deposit Stellite-6 layers over the SS410 substrate. Later, Li et al. [17] fabricated thin-walled squares of Stellite-6 on an SS304 substrate using wire arc additive manufacturing (WAAM). Subsequently, Mostafaei et al. [18] produced Stellite-6 builds using non-beam-based binder jet additive manufacturing and studied its sintering characteristics and post-heat treatment. In later years, several attempts were made to fabricate this alloy using WAAM in the form of coating over different substrates to evaluate its wear resistance for hard-facing applications [19,20,21,22,23,24] and as a single-wall build to evaluate its mechanical properties [25,26]. Also, Moradi et al. [27,28] studied the effect of processing parameters for powder-based DED fabrication of Stellite-6, such as laser power and scanning strategy.

To the best of our knowledge, no work has been reported in the open literature for fabricating Stellite-6 alloy using the laser powder bed fusion (LPBF) technique. Therefore, Stellite-6 was fabricated using LPBF and attached with a heating module for the first time in this work. The build plate was heated to above 400 °C to avoid cracking during the deposition of Stellite-6. The optimum printing parameters were identified through the dimensions of the melt pool of the single tracks to print the Stellite-6 cubes. Due to the presence of gas pores in the cubes printed using the optimized parameters, hot isostatic pressing (HIP) was performed to collapse the pores, relieve residual stresses, and homogenize the microstructure. A marked difference in the recrystallization behavior and the coarsening of the M_7_C_3_ particles was observed after HIP, which can be attributed to the differences in the grain structure and residual stresses owing to the processing parameters. Moreover, the hardness of the HIPped and wrought Stellite-6 samples is compared to evaluate the effect of HIP on the properties of Stellite-6. This work will serve as a guideline for the post-processing design of the Stellite-6 alloy fabricated using the LPBF technique.

## 2. Materials and Methods

### 2.1. Experimental Details

**Figure 1:**
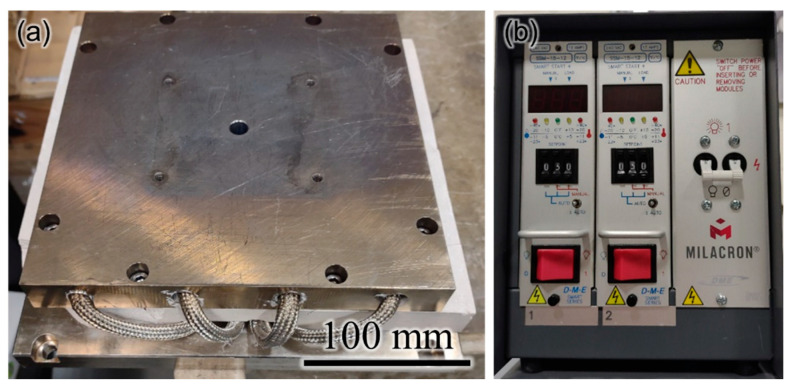
(**a**) Heating module plate showing the cartridge heaters and the ceramic plate and (**b**) the control unit.

Gas-atomized pre-alloyed powders of Stellite-6 alloy were procured from Kennametal Inc. (Pittsburgh, PA, USA), with a composition (in wt.%) of C: 1.2, Cr: 28.3, Fe: 1.8, Ni: 2, Si: 1.2, W: 4.5, O: 0.015, and N: 0.081. The powder particles comprised a bimodal distribution of sizes (45 and 10 μm) as mentioned by the vendor. Single-track prints of Stellite-6 on a substrate of the same material were performed for different combinations of laser power (250–350 W, in steps of 25 W) and scanning speed (500–1500 mm/s, in steps of 250 mm/s) using an EOS M290 machine (EOS, Krailling, Germany). The selection of the processing window was made based on standard EOS parameters for CoCr alloy (laser power = 290 W, scanning speed = 950 mm/s, and hatch distance = 110 μm). The Stellite-6 alloy contains a substantial amount of tungsten, whose ductile-to-brittle transition temperature (DBTT) is close to 400 °C. To prevent the material from going through this transition during the deposition, a high preheat temperature of 400 °C for the substrate was used. The high preheat also helps to alleviate the development of residual stress in the printed cubes. The external heating module consists of a steel plate with four cartridge heaters embedded into it. A ceramic plate is used to isolate the heating module from the rest of the machine to protect its components. Thermocouples are embedded into the cartridge heaters and hooked into the controller, as shown in Figure 1. After identifying the two optimal combinations of laser power and scanning speed based on an analysis of the melt pool characteristics, cubes of 15 × 15 × 15 mm^3^ dimension were printed using those parameters with different hatch spacing and layer thickness. The total printing time for eight cubes of 15 × 15 × 15 mm^3^ with varying parameters of processing that we tested was about 50 min, of which only two were successful without cracks. Including other factors, such as setting up the heating module, heating the build plate, and cooling before the build can be removed from the machine, it took around four hours to finish printing eight cubes. A hatch spacing of 72 μm and layer thickness of 40 μm was maintained during the printing. The substrate was heated to 450 °C for printing the cubes to ensure that the preheat was high to avoid cracking while building multiple layers. The samples were subjected to HIP at 1250 °C for 2 h with a pressure of 150 MPa in an AIP6-45H Mo furnace (American Isostatic Presses Inc., Columbus, OH, USA). The heating rate was 15 °C/min, and after applying HIP, the sample was cooled using drop cooling by switching off the furnace. The initial cooling rate was 40 °C/min up to around 700 °C, and then it slowed down. The total cooling time was 1 h. An argon atmosphere was used for the HIP of the Stellite-6 builds.

The printed Stellite-6 cubes were extracted from the build plate using electric discharge machining. The cross-section of the melt pool from the single-track prints was viewed under a Zeiss Axio Lab A1 optical microscope (Jena, Germany), and the ImageJ software (version 15.3) was used to evaluate the melt pool width and depth. Microstructure characterization was performed in the XZ plane (X—scanning direction and Z—build direction) of the build. A scratch-free surface with a mirror finish was prepared by grinding from 600 to 1200 grit SiC papers, followed by polishing with diamond (3 and 1 μm) and silica (0.04 μm) suspensions. The complete surface of the build before and after HIP was stitched from multiple images captured using the Zeiss Smartzoom 5 automated digital optical microscope. The as-polished surface of the builds before and after HIP was imaged using a FEI Scios Dual Beam focused ion beam–scanning electron microscope (SEM) attached to a field emission gun source. The composition of the phases was measured using an OctaneElite energy-dispersive spectroscope (EDS) system attached to the SEM. Electron backscattered diffraction (EBSD, EDAX Hikari EBSD system, Berwyn, PA, USA) attached to the SEM was used for further detailed phase and grain structure analysis of the Stellite-6 builds. The microhardness measurements were performed using an automated Vickers hardness tester (AMH55 with LM310AT Microindenter, St. Joseph, MI, USA) with a load of 300 g and a dwell time of 10 s. The reported values are an average of 20 readings.

### 2.2. Computational Details

**Figure 2:**
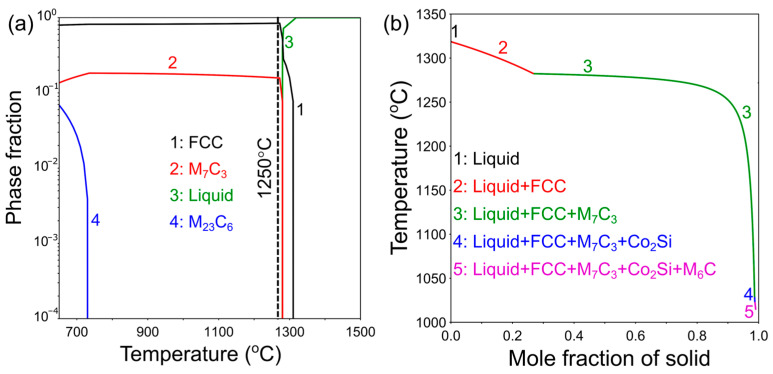
(**a**) Equilibrium phase fraction as a function of temperature and (**b**) Scheil solidification path calculated using TCFE11 database for the Stellite-6 feedstock composition.

The equilibrium and Scheil calculations were performed using Thermo-Calc software (version 2021b). The composition of the powder feedstock was used as input for all the simulations. The commercial multicomponent thermodynamic database for steels (TCFE11) was used for the equilibrium and Scheil calculations. The commercial thermodynamic database for steels (TCFE) was used in this work instead of the Ni-based superalloy database (TCNI), which is generally used for Co-based alloys because the TCFE databases can predict the carbides efficiently. Since Stellite alloys are Co-based alloys with Cr-rich carbides with a maximum content of ~80 mol% as secondary phases, the TCFE database was expected to predict the phase equilibria more accurately than TCNI. In the Scheil simulation, carbon was considered to be a fast-diffusing species. The equilibrium phase fraction plot and the Scheil solidification diagram calculated for the Stellite-6 powder composition are shown in Figure 2.

## 3. Results and Discussion

**Figure 3:**
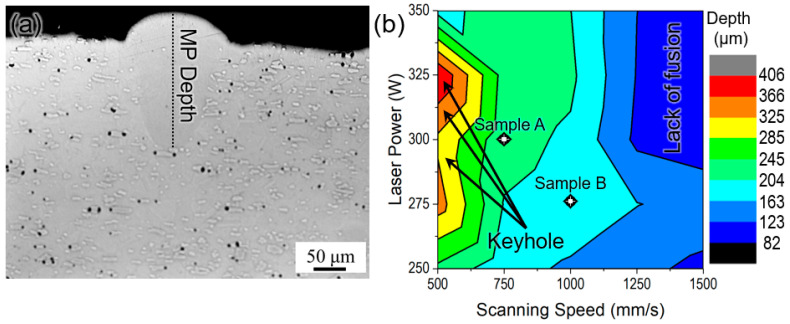
(**a**) A representative optical micrograph of the single-track print with external heating of the build plate at 400 °C showing the marked melt pool depth and (**b**) a contour plot as a function of laser power and scanning speed for melt pool depth showing the regions identified for keyholing and lack of fusion. Samples A (300 W/750 mm/s) and B (275 W/1000 mm/s) denote the optimum parameters chosen for printing the Stellite-6 cubes.

A representative optical micrograph of the cross-section of the single track with the melt pool depth marked is shown in Figure 3a. As mentioned earlier, the temperature of the build plate while printing the single track is 400 °C, which was achieved using an external heating module. The variation in melt pool depth as a function of laser power and scanning speed is shown in Figure 3b. It can be clearly observed from the contour plot that a higher scanning speed will lead to a lack of fusion with a lower melt pool depth (82–163 μm). On the contrary, lower scanning speed will lead to keyholing with a deeper melt pool (285–406 μm) for all laser powers (except 350 W), which is undesirable. With a laser power of 350 W, the melt pool depth did not fall into the regime of keyholing; however, the melt pool was wide (>200 μm). With the formation of a large melt pool, a higher amount of evaporation from the molten material is possible [29]. Since chromium is more prone to evaporation due to its low vapor pressure, it is not feasible to use a laser lower than 350 W with a slower scanning speed, leading to the formation of a wider melt pool. The optimum combination lies in the middle portion of the contour plot, and it is desired to have an optimum melt pool depth (160–240 μm). Therefore, two combinations of laser power and scanning speed were chosen for the printing of the cubes, as listed in Table 1.

**Figure 4:**
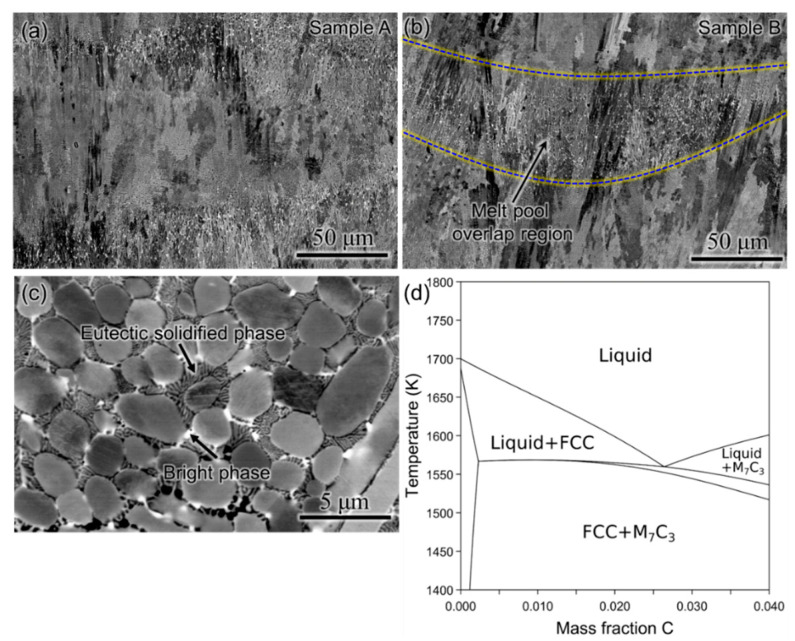
(**a**) SEM micrographs in secondary electron mode for (**a**) sample A and (**b**) sample B (melt pool overlap region marked with blue dashed lines), (**c**) a high-magnification SEM micrograph from the melt pool overlap region showing primary FCC phase in dendritic and equiaxed forms along with bright and eutectic solidified (with light and dark layers) phases in the interdendritic regions, and (**d**) calculated vertical section with the variation in carbon as a function of temperature (at constant Cr and W content) showing the eutectic line (L → FCC + M_7_C_3_), and hence, the eutectic solidified phase region in (**c**) comprises FCC (light layer) + M_7_C_3_ (dark layer) phases.

Figure 4a,b present the SEM micrographs from samples A and B captured in secondary electron mode. The microstructure consists of clearly visible dendrites throughout the build, and the overlapping regions between the melt pool (marked with blue dashed lines in Figure 4b) are found to have a different structure than the interior of the melt pool in both the builds. From the high-magnification SEM micrograph from the melt pool overlapping region (Figure 4c), it is evident that apart from the dendritic and equiaxed grains of the primary phase, bright phases and several dark phases with alternate layered eutectic structure were found in between these grains. The non-equilibrium solidification path predicted using the Scheil module of Thermo-Calc software using the TCFE11 database for the feedstock composition is shown in Figure 2b. The FCC phase rich in Co and Cr is the primary solidifying phase, which is the phase with equiaxed grains as displayed in Figure 4c. According to Guyard et al. [27], during the solidification of Stellite-6 alloys, the liquid meets a eutectic line with the co-precipitation of the FCC and M_7_C_3_ phases. In addition, a vertical section was calculated using the TCFE11 database of Thermo-Calc software at constant Cr and W content with varying composition of carbon as a function of temperature, as shown in Figure 4d. It can be observed that a eutectic monovariant reaction (L → FCC + Cr_7_C_3_) occurs, which is in agreement with the statement by Guyard et al. [30]. Thus, a eutectic solidified phase with alternating light and dark layers can be observed in the interdendritic region, with the dark layer corresponding to Cr_7_C_3_ and the light layer indicating the FCC phase, as seen in Figure 4c.

**Figure 5:**
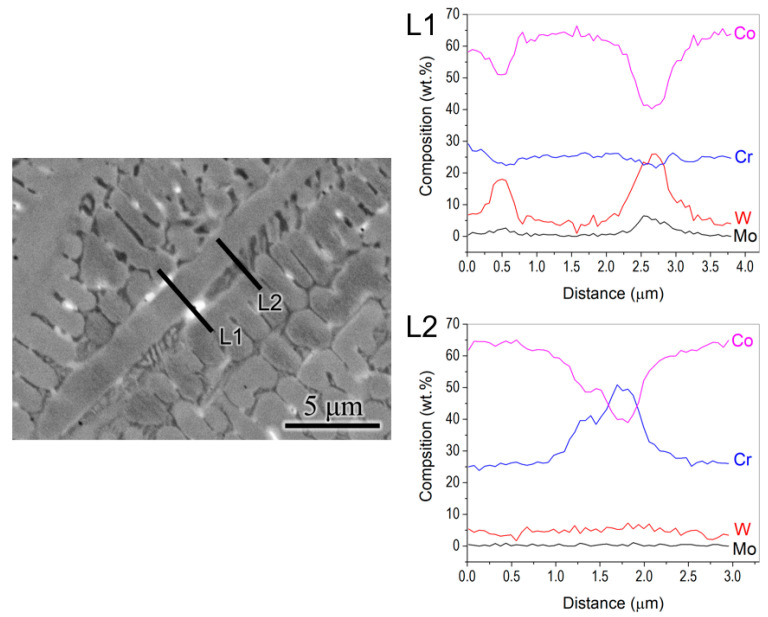
EDS line scans from the interdendritic regions with bright and dark phases, showing that the bright phase (L1) is rich in tungsten (W-rich M_6_C phase), whereas the dark phase (L2) is rich in chromium (Cr_7_C_3_ phase).

In order to probe further, the phases formed between the primary phase grains in the overlapping regions between the melt pool, and EDS line scans were performed, as shown in Figure 5. The bright phases were rich in W, while the dark phases were rich in Cr. Moreover, from the Scheil simulation (Figure 2b), the final phase to solidify is M_6_C, which is rich in W, whereas the first secondary phase to form in the FCC matrix is M_7_C_3_, which is rich in Cr. Therefore, the bright phase is identified as the M_6_C phase, while the dark phase corresponds to M_7_C_3_ in the interdendritic regions.

**Figure 6:**
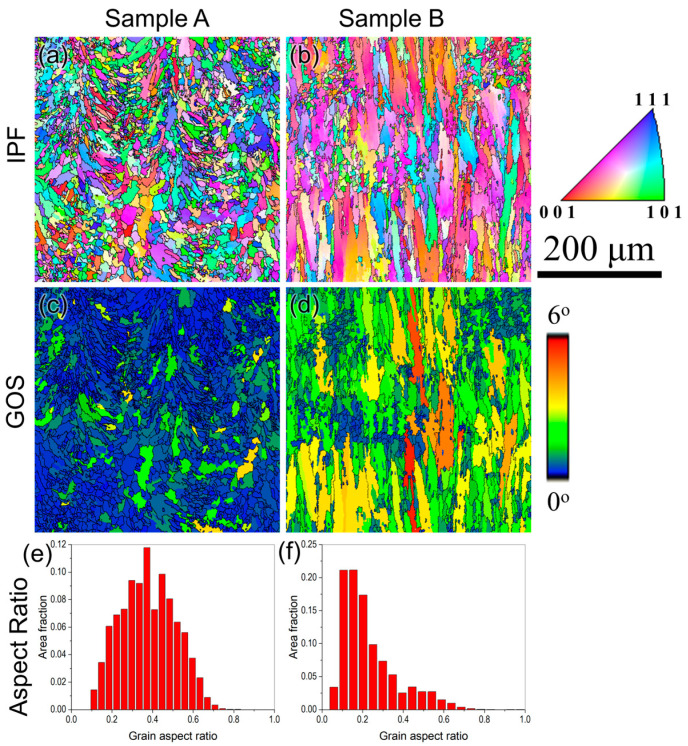
(**a**,**b**) Inverse pole figure maps, (**c**,**d**) grain orientation spread maps, and (**e**,**f**) grain aspect ratio as a function of area fraction for samples A and B obtained using EBSD in as-built condition showing the difference in the grain structure of these two builds.

Further analysis of the as-built microstructure for samples A and B was performed using EBSD, as shown in Figure 6. From the inverse pole figure (IPF) maps (Figure 6a,b) superimposed with the high-angle grain boundaries (>15°), it can be observed that the grains are finer and equiaxed in sample A, while they are elongated in sample B. Moreover, for the same magnification, several melt pool boundaries can be seen in sample A, whereas only one partial melt pool boundary can be observed in sample B, indicating that the melt pools in sample B are larger than in sample A. Moreover, the grain structure of sample B is highly elongated compared to sample A. The grain orientation spread (GOS) maps give a qualitative idea about the residual stress in a build, where lower GOS indicates lower residual stresses and vice versa. It is defined as the ratio between the mean value of misorientations between all the pixels of the grain and the mean orientation of the grain [31]. The GOS value within each grain denotes the degree of distortion. A larger grain distortion signifies higher stored energy, i.e., residual stress within a grain, which results in a higher GOS value [32]. It is clearly evident that the residual stress is lower in sample A with lower GOS (higher fraction of blue regions with 0° GOS) in comparison with sample B with higher GOS (higher fraction of green and yellow regions with GOS between 2 and 4°), as can be seen from Figure 6c,d.

The residual stresses in sample A are expected to be lower than in sample B since the scanning speed during the printing of this sample is slower (750 mm/s) and the laser power is higher (300 W). According to Vrancken [33], using high heat inputs reduces the residual stresses that include factors such as high laser powers, slower scanning speeds, thin layer thickness, and preheating the base plate. In the current scenario, since both samples A and B were preheated to the same temperature (450 °C), the residual stresses in sample A will be lower since it was printed with higher laser power and lower scanning speed in comparison with sample B. Similarly, the aspect ratio of the grains is also strikingly different between these two builds, with sample A having higher value (average ~0.45), indicating a lower fraction of elongated grains, compared to a lower value (average ~0.2) for sample B, denoting a highly elongated grain structure, as shown in Figure 6e,f.

**Figure 7:**
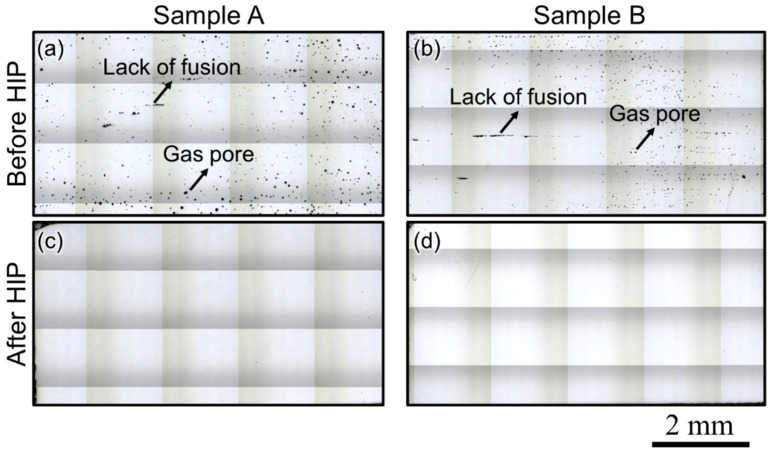
Macrostructure of the cross-section of samples A and B (**a**,**b**) before hot isostatic pressing (as-built condition) and (**c**,**d**) after hot isostatic pressing, showing that the HIP has effectively mitigated the porosity in the builds.

After the detailed microstructure analysis for the two builds of Stellite-6 successfully printed using LPBF, the analysis of the macrostructure of the cross-section of the build was performed, as shown in Figure 7. Figure 7a,b show the presence of gas pores and lack of fusion pores in both builds. The presence of gas pores can be attributed to the presence of oxygen and nitrogen in the powder feedstock, which can escape during the printing and, hence, lead to the formation of tiny spherical gas pores throughout the cross-section of the build. The lack of fusion pores was mainly observed at the interlayer boundaries of the build, and hence, it can be attributed to the development of high thermal residual stresses during the printing process, even with a preheated build plate. Since the as-built microstructure of samples A and B is elongated along the build direction, it will lead to anisotropy in the properties. Thus, a recrystallized microstructure will be essential for achieving isotropic properties in the Stellite-6 build. The presence of pores and an anisotropic microstructure necessitates the application of HIP to both builds.

Therefore, HIP needs to be applied to the Stellite-6 builds printed using the LPBF technique to mitigate porosity, recrystallize the microstructure, and relieve the residual stresses developed during printing. The standard solution treatment temperature reported for Stellite-6 is 1200 °C, followed by air cooling [34]. However, Ren et al. [15] performed the solution heat treatment at 1200 °C for 1 h for Stellite-6 manufactured using powder-based DED, whereas Mostafaei et al. [18] could achieve 99.8% density after sintering at 1310 °C for 1 h for binder jet additively manufactured Stellite-6 builds. In addition, from the equilibrium phase fraction plot presented in Figure 2a, no secondary phases were present except the M_7_C_3_ between 750 and 1280 °C, and it is not possible to dissolve M_7_C_3_ completely without melting the matrix (FCC) phase. Thus, the HIP temperature was chosen as 1250 °C with a pressure of 150 MPa. The holding time was chosen as 2 h, which is an hour longer than the commonly used homogenization time to ensure complete closure of the pores along with recrystallization during HIP. The macrostructures of samples A and B after HIP (Figure 7c,d) show a complete pore closure, indicating that the HIP treatment was effective in mitigating the porosity present in the as-built Stellite-6 builds fabricated using the LPBF technique.

**Figure 8:**
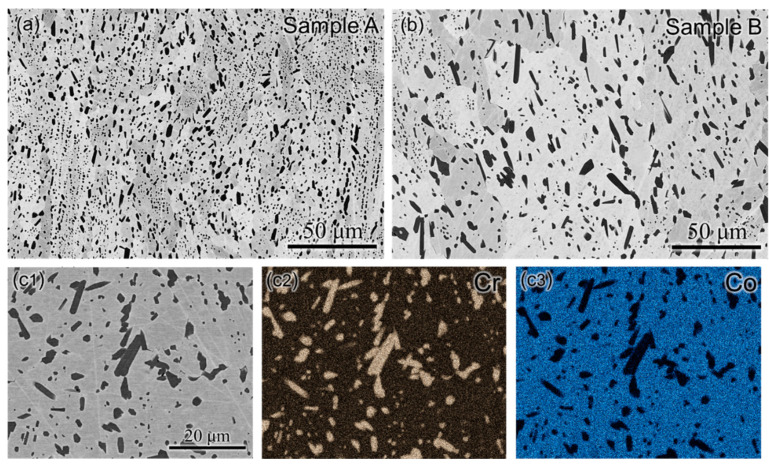
SEM micrographs in secondary electron mode after HIP for (**a**) sample A and (**b**) sample B showing a gray matrix phase with dispersed black secondary phases. (**c1**) SEM micrograph, (**c2**) Cr map, and (**c3**) Co map showing that the dispersed black secondary phases are rich in Cr and devoid of Co, and hence, it corresponds to M_7_C_3_ particles.

The SEM micrographs captured in secondary electron mode after HIP for samples A and B show a gray matrix phase with black precipitates dispersed throughout the matrix, as presented in Figure 8a,b. The dendritic structure with the overlapping melt pools observed in the microstructure of as-built samples A and B completely disappeared after HIP. The composition of the precipitates obtained using the EDS area scan (Figure 8(c1)–(c3)) exhibits that the dispersed black phases are rich in Cr and devoid of Co; hence, they are identified as M_7_C_3_ particles. Therefore, the phase that was segregated in the interdendritic regions of the as-built sample has transformed into a dispersed secondary phase after HIP in both samples. The W-rich M_6_C phase that was found in the melt pool overlapping regions dissolved into the matrix after HIP. Moreover, it is clearly evident that there is a marked difference in the coarsening behavior of the M_7_C_3_ particles in samples A and B. The size, volume fraction, and number density of the M_7_C_3_ particles after HIP were measured using ImageJ software from the SEM micrographs. For sample A, the size, volume fraction, and number density of the M_7_C_3_ particles were measured as 0.85 ± 0.21 μm, 0.149 ± 0.015, and 1.25 × 10^18^ ± 3.31 × 10^17^, respectively. Though the volume fraction (0.144 ± 0.011) of these particles was similar for samples A and B, the size (2.88 ± 0.78 μm) and number density (3.76 × 10^17^ ± 1.23 × 10^17^) were markedly different. This proves that during the application of HIP using the same parameters for the two Stellite-6 builds fabricated by the LBPF technique using different processing parameters, the M_7_C_3_ particles in sample B coarsened faster than those in sample A.

Similar observations have been reported for Ni-based superalloys manufactured using LPBF, which showed coarsened Cr-rich carbides after HIP [35,36]. Due to the high heating and cooling rates during LPBF processing, sufficient time is not available for Cr to diffuse fully and form blocky carbides. Therefore, tiny regions of Cr-rich carbide can be observed in the interdendritic regions, and the remaining Cr is trapped in the matrix of the as-built Stellite-6 builds. During HIP at 1250 °C, re-segregation of Cr occurs at the grain and sub-grain boundaries, and hence, blocky M_7_C_3_ carbides can grow in these regions. Moreover, due to the slow cooling rate after HIP, there is sufficient time available for the carbides to grow further. This explains the presence of blocky M_7_C_3_ carbides observed after HIP in samples A and B. In addition, it has been reported that a higher scanning speed will result in a higher solidification rate. Thus, the extent of microsegregation can be suppressed by solute trapping [37], leading to higher supersaturation of alloying elements in the matrix. Since sample B (1000 mm/s) was printed with a higher scanning speed than sample A (750 mm/s), a lesser extent of microsegregation and higher supersaturation of Cr in the matrix is expected in sample B. For the same holding time during HIP, sample B has more Cr content in the matrix than sample A, making the carbides possibly nucleate quicker due to faster diffusion of Cr entering into the growth regime, leading to excessive coarsening of the M_7_C_3_ carbides in sample B.

**Figure 9:**
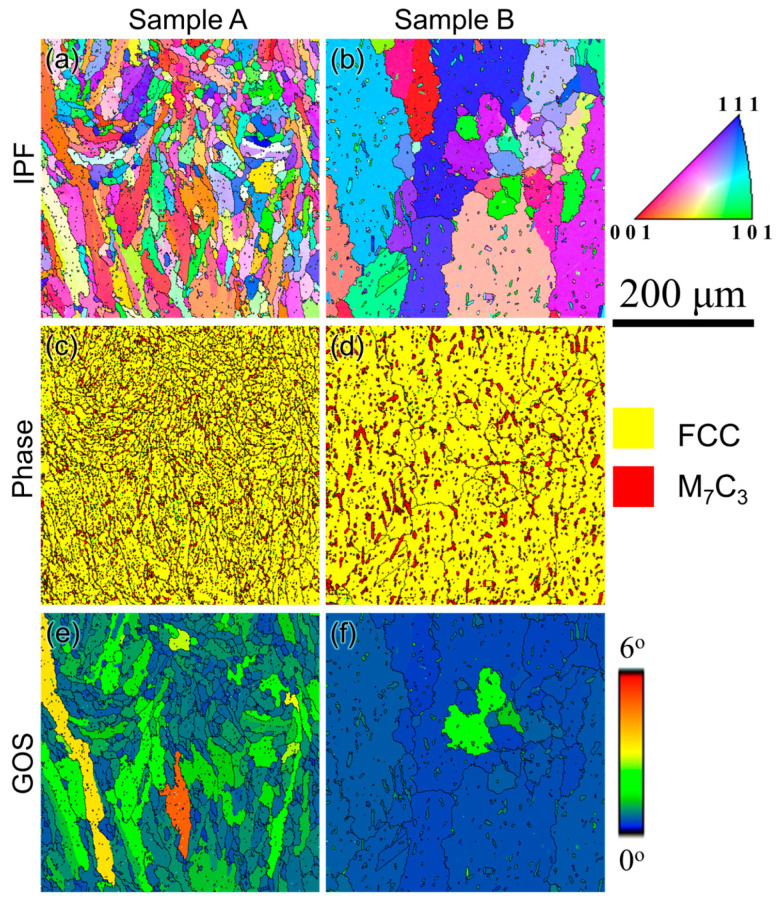
(**a**,**b**) Inverse pole figure maps, (**c**,**d**) phase maps, and (**e**,**f**) grain orientation spread maps for samples A and B obtained using EBSD showing the difference in the grain structure and carbide coarsening behavior of these two builds after HIP.

The IPF, phase, and GOS maps superimposed with high-angle grain boundaries (>15°) for samples A and B after HIP are shown in Figure 9. It can be observed that there is excessive grain coarsening along with complete recrystallization of grains after HIP in sample B, while only a partial recrystallization with negligible coarsening is found in sample A. From the phase maps (Figure 9c,d), it is clearly evident that a mixture of coarse and fine carbides (red phases) is observed in sample A, where the coarse carbides are predominantly found in the grain boundaries, while the fine carbides are mostly found within the grains. On the other hand, coarse carbides are found throughout the matrix in sample B. Grains with GOS less than 2° can be considered as recrystallized grains [38]. The GOS maps (Figure 9e,f) show that sample A has a higher fraction of grains with GOS > 2° (yellow and orange regions), while sample B has no grain with GOS > 2°. The fraction of recrystallized grains was 86% in sample A and 100% in sample B. One of the main driving forces for the recrystallization of grains is the stored energy in the form of residual stresses. Therefore, the faster recrystallization followed by grain growth in sample B can be mainly attributed to the higher residual stresses in the as-built condition, which can be clearly observed from the grains with high GOS (3° < GOS < 5°) in Figure 6d. The grains with predominantly lower GOS in as-built sample A (Figure 6c) indicate that the driving force was insufficient, suggesting that partial recrystallization has occurred. In addition, the elongated grains with a lower mean grain aspect ratio in sample B are also expected to induce faster recrystallization.

**Figure 10:**
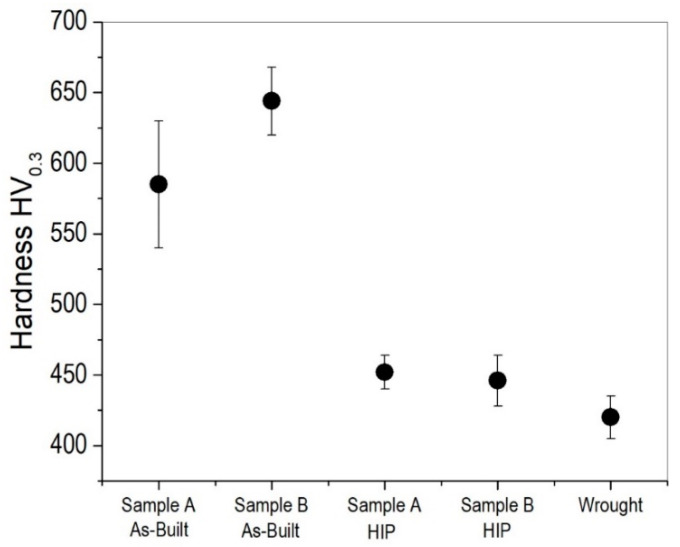
Microhardness of samples A and B in as-built and HIP conditions compared with wrought Stellite-6 plate.

The microhardness of Stellite-6 samples A and B in as-built condition and after HIP, along with a comparison with its wrought counterpart, is shown in Figure 10. The microhardness of as-built sample B is higher than that of sample A by nearly 50 HV, which proves that the residual stresses are higher in the former than the latter, though it is the same material with a similar microstructure. After HIP, the hardness is reduced by approximately 200 HV, which is mainly because of the relief in the residual stresses during HIP. Though there is significant coarsening of the M_7_C_3_ phase in sample B after HIP, its effect is not visible in the hardness. It is worth noting that the hardness of samples A and B is slightly higher (20 HV) than the hardness of the wrought Stellite-6 plate. This proves that the application of HIP did not have an adverse effect on the hardness of Stellite-6 fabricated using LPBF, and it is on par with its wrought counterpart. However, the grain growth and carbide coarsening are expected to have an adverse effect on the wear resistance of this alloy, which is a potential area for future work.

From these observations, it can be understood that the processing parameters significantly affect the recrystallization behavior during the HIP of Stellite-6 alloy fabricated using the LPBF technique. There is scope to modify the grain structure HIP with a subsequent solution heat treatment. In addition, based on the literature report, secondary heat treatments after HIP can increase porosity since the high internal pressure in the retained negligible fraction of pores can act as the driving force for them to grow [31]. This proves that HIP needs to be explicitly designed for builds fabricated with different processing parameters for Stellite-6 manufactured using LPBF with an external heating module. Applying the same HIP parameters for all builds fabricated with different laser powers and scanning speeds can partially recrystallize one build, whereas it can induce grain growth in another build.

## 4. Conclusions

For the first time, crack-free Stellite-6 samples were fabricated using the LPBF technique equipped with an external heating module. Single-track prints with a preheat of 400 °C were employed for identifying the optimum processing parameters with ideal melt pool depth (185–200 μm). Two crack-free cubes (sample A: 300 W and 750 mm/s and sample B: 275 W and 1000 mm/s) were printed successfully. However, several spherical gas pores and lack of fusion pores were observed. The as-built microstructure consisted of the FCC matrix with columnar grains and the M_7_C_3_ phase in the interdendritic region. The melt pool overlapping regions consisted of the W-rich M_6_C phase, supported by the Scheil calculation. The eutectic phase with alternating layers of FCC and M_7_C_3_ phases was observed due to a eutectic reaction (L → FCC + M_7_C_3_) as predicted by thermodynamic calculation. Sample A consisted of grains with lower GOS, whereas sample B had grains with higher GOS, implying that the residual stress in sample B was higher than in sample A. As a result, sample A was partially recrystallized with a mixture of slightly coarse and fine M_7_C_3_ particles after HIP. On the contrary, sample B underwent complete recrystallization with grain growth due to the higher residual stress in as-built condition. Significant coarsening of M_7_C_3_ was also observed, possibly due to the higher Cr supersaturation in the matrix and the higher scanning speed for sample B. The hardness of sample B was 50 HV higher than sample A in as-built condition, which proves that the residual stress in sample B is higher. The hardness reduced drastically after HIP due to the release of the residual stresses; still, they were slightly higher than their wrought counterparts, proving that there is no adverse effect of HIP on the properties of Stellite-6 alloy.

## Figures and Tables

**Table 1:** Processing parameters identified for printing the Stellite-6 cubes using LPBF based on their melt pool depth from the single-track prints.

	Laser Power (W)	Scanning Speed (mm/s)	Energy Density (J/mm^3^)	Melt Pool Depth (μm)
Sample A	300	750	139	209
Sample B	275	1000	95	186

## Data Availability

The original contributions presented in the study are included in the article; further inquiries can be directed to the corresponding authors.
